# Steady and Oscillatory Shear Flow Behavior of Different Polysaccharides with Laponite^®^

**DOI:** 10.3390/polym13060966

**Published:** 2021-03-22

**Authors:** Marcos Blanco-López, Álvaro González-Garcinuño, Antonio Tabernero, Eva M. Martín del Valle

**Affiliations:** 1 Department of Chemical Engineering, University of Salamanca, Plaza los Caídos s/n, 37008 Salamanca, SA, Spain; marcosbl97@usal.es (M.B.-L.); alvaro_gonzalez@usal.es (Á.G.-G.); 2 Instituto de Investigación Biomédica de Salamanca, Hospital Virgen de la Vega, Paseo San Vicente, 58-182, 37007 Salamanca, SA, Spain

**Keywords:** Laponite^®^, polysaccharides, levan

## Abstract

The rheological behavior, in terms of steady and oscillatory shear flow, of Laponite^®^ with different polysaccharides (alginate, chitosan, xanthan gum and levan) in salt-free solutions was studied. Results showed that a higher polymer concentration increased the zero-rate viscosity and decreased the critical strain rate (Cross model fit) as well as increasing the elastic and viscous moduli. Those properties (zero-rate viscosity and critical strain rate) can be a suitable indicator of the effect of the Laponite^®^ on the shear flow behavior for the different solutions. Specifically, the effect of the Laponite^®^ predominates for solutions with large critical strain rate and low zero-rate viscosity, modifying significantly the previous parameters and even the yield stress (if existing). On the other hand, larger higher polymeric concentration hinders the formation of the platelet structure, and polymer entanglement becomes predominant. Furthermore, the addition of high concentrations of Laponite^®^ increases the elastic nature, but without modifying the typical mechanical spectra for polymeric solutions. Finally, Laponite^®^ was added to (previously crosslinked) gels of alginate and chitosan, obtaining different results depending on the material. These results highlight the possibility of predicting qualitatively the impact of the Laponite^®^ on different polymeric solutions depending on the solutions properties.

## 1. Introduction

The Clay Minerals Society defines clay minerals as “minerals which impart plasticity to clay and which harden upon drying or firing” [1]. One of the main characteristics that defines these minerals is its sheet silicate crystal structures, varying in dimensions and compositions, due to their purely natural origin, affected by the geological context. Phyllosilicate clay materials present high sorption capability, swelling capacity, surface area and reactivity to acids which makes them appropriate for use in pharmaceutical and cosmetic products. More concretely, the smectite group within phyllosilicate clay materials has direct applications as both active principles, such as gastrointestinal protectors, and excipients, like diluents or emulsifying [2].

Among these clays, Laponite^®^ is found to be a synthetic clay mineral with structural similarities to hectorite. These phyllosilicates are structured by 2:1 crystal with layered units composed of two tetrahedral silica sheets surrounding a magnesium cation (Mg^2+^) containing octahedral sheet. These crystals are organized in disks conformation, with approximate dimensions of 25 nm as diameter and 0.92 nm thick. Its empirical formula Na^+0.7^[(Si_8_Mg_5.5_Li_0.3_)O_20_(OH)_4_]^−0.7^ implies substitution of magnesium cations for lithium ones. This substitution provokes the faces of the disks to be negatively charged in order to keep stability with the cations they are establishing ionic/covalent chemical bonds. These interactions and the ones with the water molecules located nearby result to be electrostatic, forming crystal stacks as dry powder texture. This clay can be expanded with the water to compensate the weak negative charge of the disk faces, developing a volume increase. When it comes to edges charge, the hydroxyl groups that can be protonated varying the environmental pH must be taken into account, finding them to be 11 for positively charged edges [3]. Physical properties are stated as 2.53 g/cm^3^ for density and 5.2 × 10^17^ disks/g for a 1.5 nm layer periodicity [4].

Laponite^®^ compressed disks experiment an appreciable change when in aqueous systems. The electrostatic forces that retain its disks together when dried start repelling amongst them, causing the individual crystals to separate forming colloidal dispersions. These crystals may interact with others, forming different arrangements depending on the repulsive and attractive interactions. Amongst these structures, at low ionic strength and specific concentrations, a structure known as “House of cards” can be favored [5]. In this context, structural evolution of Laponite^®^ dispersion and aging kinetics have been investigated in [6].

Due to its unique structure, Laponite^®^ has been proposed as a carrier for different drug delivery systems. Drug inclusion can be produced by adsorption on the clay surface (interactions with the negatively charged surfaces or positive charged edges) or by intercalation inside the interlayer space [7]. More information about how to intercalate small organic molecules and polymers within smectite crystal stacks can be found elsewhere [8,9].

Moreover, Laponite^®^ formation in aqueous solutions can work as protection of drugs against degradation in the physiological environment. This phenomenon was showed by Park et al. [10], who mixed donepezil (drug used for Alzheimer’s disease treatment) with Laponite^®,^ and by Jung et al., who prepared a composite of Laponite^®^ XLG and itraconazole [11]. Both studies focused on drug delivery in human gastric simulation media. Laponite^®^ XLG was found to establish interactions not only with charged species but also with polar and non-polar compounds.

Other properties that promote its use on biomedical applications are for instance, its degradation and cytotoxic behavior. Laponite^®^ under acidic conditions promote the release of degradation products such as aqueous silica Si(OH)_4_, sodium, lithium and magnesium ions to the medium [12]. When it comes to cytotoxic behavior, Laponite^®^ XLG is stated as non-toxic when concentrations below 1 mg/mL are used [13].

Therefore, Laponite^®^ can be considered as a source for the design of new biomedical materials with a higher biological performance, also when it comes to nanoscale. One of the most important and primal uses was its function as a supporting compound for preparing hydrogels. The properties conferred by its clay structure enable the interaction with a wide range of molecules making it a suitable component in drug delivery systems, as well as regenerative micro-environment sites [9].

Particularly, Laponite^®^ has been used with polysaccharides to produce composites for biomedical applications. Polysaccharides are biocompatible materials with a strong natural character and with special advantages for their use in humans. Alginate, chitosan, xanthan gum or levan are well-known polymers that have been used for developing drug delivery systems, with adequate properties for biomedicine [14,15].

The addition of Laponite^®^ improved polysaccharides’ material properties. Doxorubicin was loaded in a pH sensitive hydrogel alginate-Laponite^®^, demonstrating a high drug loading capacity and displaying a sustained release profile of the drug that could be used for a prolonged antitumor therapy. Moreover, the complex was able to overcome the physiological doxorubicin resistance through ion trapping inside acidic compartments [16]. Transport through cell membranes is highly efficient, and Laponite^®^ presence promotes these complexes as promising vectors for the delivery of weak bases chemotherapeutics or even oral drugs for stomach diseases. Laponite^®^ was also processed with chitosan to develop a scaffold for wound healing applications. The material had an increase in porosity and an improvement in the mechanical properties after the addition of the clay [17]. Laponite^®^ was added to a gellan gum’s injectable hydrogel to confer stability and strength to the obtained material [18]. That fact was promoted due to the above-mentioned clay-polymer interactions that increased the elastic modulus. Therefore, controlling the effect of the Laponite^®^ in the material′s rheological properties is a key parameter to know the final properties of the material [18].

As a consequence, the effect of Laponite^®^ in the rheological behavior of alginate′s solution has been studied. This work showed that the effect of the Laponite^®^ depends on the alginate concentration and that these solutions experience an aging phenomenon. The house of cards structure can be formed for low initial concentrations, forming a gel by means of a physical gelation due to clay-polymer interactions. However, achieving the house of cards structure is more difficult for high alginate concentrations [19].

When it comes to xanthan gum solutions in salt solutions, Laponite^®^ effect makes its rheological behavior change [20]. Again, the effect is depending on the polymer concentration. Entanglement in this system is based on complex structures in polymeric matrices, including interactions between polymer chains, clay particles and between particles and chains. Moreover, Laponite^®^ dispersions mixed with a polysaccharide gel, such as scleroglucan, strongly depend on concentration [21]. However, the previous results were dedicated to individual systems Laponite^®^-polymer, without studying the effect of the Laponite^®^ on different polysaccharides depending on polymer solution properties.

Therefore, the effect of the Laponite^®^ in polymeric solutions depends on nanoclay-polymeric interactions, modifying the structural conditions of both components. However, it is still unknown if it is possible to connect the effect of the Laponite^®^ on the rheological properties (in terms of oscillatory and steady flow behavior) with polysaccharide conformation in solutions and polymeric solutions characteristics, such as pseudoplasticity, zero-rate viscosity or critical strain rate. Based on the previous facts, this work proposes the study of the different rheological parameters for different Laponite^®^-polysaccharides mixtures and their forming gels. The Laponite^®^ effect will be studied in solutions of different natural-origin polysaccharides, such as alginate, xanthan gum, levan and chitosan. These polymers were selected because they have different structures, rheological behavior and different ionic charge. Steady and oscillatory flow experiments will show the effect of Laponite^®^ on their solutions properties. The obtained results will be useful for future applications concerning the development of systems polysaccharides-Laponite^®^ and can provide some information about the effect of the Laponite^®^ on different polysaccharides solutions.

## 2. Polysaccharides

Firstly, the structure of the different polysaccharides, which will be studied with Laponite^®^, will be summarized.

Xanthan gum is a hetero polysaccharide which is synthetized by fermentation of the *Xanthomonas campestris* bacterium. Its backbone is structured in beta,1-4-D-glucose with three chains consisting of two mannose residues separated by glucuronic acid in every alternate glucose unit. The mannose molecule closest to the backbone can carry an acetyl group, whilst the terminal one may carry a pyruvate one. A native ordered and rigid conformation has been reported to exist as a double strand helix, even though a single helical conformation can also be present. The structure and physical properties stability depend on the ionic environment, which could significantly affect it. Moreover, different studies indicated that a yield stress parameter should be included to obtain an appropriate fit of the experimental data [22,23].

Alginate is an anionic polysaccharide extracted from macroalgae or bacterial cultures. It is a copolymer based off 1-4 linked beta-D-mannuronate (M) and alpha-L-guluronate (G) residues. In this case, the ratio between both of the residues is affected by the source. Alginate is a block polymer, characterized by linear and unbranched copolymers containing similar or strictly alternating blocks: MM, GG acids covalently linked forming different sequences. Two adjacent G blocks can be cross-linked with multivalent cations: divalent ones, for instance, Ca^2+^, Ba^2+^, Fe^2+^, Sr^2+^ or trivalent cations, such as Al^3+^. When a gelling mechanism is applied, cations take part in ionic binding zones between G blocks, and thus, a three-dimensional network is synthesized [24,25].

Chitosan is a cationic polysaccharide constituted of copolymers of beta (1→4) linked glucosamine and N-acetyl-glucosamine. It belongs to the glycosaminoglycan family (GAGs). They express the property of bioactivity and are able to dilute in acidic media. Many delivery formulations based on chitosan are usually prepared by chemical cross-linking with gluteraldehyde, urea formaldehyde, etc. [15].

Levan is a fructose exopolysaccharide that is obtained after cultivating bacteria such as *Zymomonas mobilis* or Bacillus subtilis. Its structure is formed by fructose units bonded with beta (2→6) linkages with some ramifications beta (2→1) [26]. Levan is characterized by its ability to form nanoparticles in water by self-assembly, above a critical aggregation concentration, due to its amphiphilic properties [27]. Levan solutions are considered non-viscous, since they only achieve a certain degree of viscosity at high concentrations [28].

## 3. Materials and Methods

### 3.1. Materials

Xanthan gum (CAS 11138-66-2) with an average molecular weight around 1000 kDa, chitosan (CAS 9012-76-4) with an average molecular weight around 250 kDa and alginate (CAS 9005-38-3) with an average weight around 150 kDa were purchased from Sigma Aldrich, Madrid, Spain. Fructosyltransferase 68S from *Bacillus subtilis* (NATE 1384), which was used to obtain levan, was purchased from Creative Enzymes, NY, USA. Finally, Laponite^®^ XLG was obtained from BYK Additives and Instruments, Madrid, Spain.

### 3.2. Levan Obtention

Levan was obtained with a cell-free methodology (molecular weight of 2000 kDa) that was described elsewhere [27]. Basically, the enzyme was added (0.2 mg·L^−1^) to a solution of sucrose of 90 g·L^−1^ in an AFORA reactor of 100 mL at 37 °C. After 72 h, the reaction was stopped, and the polymer was isolated by adding firstly three volumes of ethanol to the reaction mixture. After that, the mixture was stored at −20 °C for 24 h to precipitate the polymer. Finally, with a centrifugation (Eppendorf Centrifuge 5804, Nussloch, Germany) during 10 min at 10,000 rpm the levan was obtained. Finally, the polymer was dried by lyophilization (Telstar, Barcelona, Spain) at −55 °C, 0.05 bar.

Levan nanoparticles size and Z-potential were measured by dynamic light scattering technique with a Zetasizer (Malvern, Malvern, UK).

### 3.3. Rheological Analysis

The rheological properties of the different systems were studied with a Rheometer AR 1500 ex (TA Instruments, Madrid, Spain), equipped with an aluminum plate (diameter 4 cm) and gap of 1 mm.

Firstly, different solutions of polymers in distilled water and solutions of polymer-Laponite^®^ (in this case both compounds were dissolved and dispersed together) were prepared, and the sample was loaded on the peltier. After that, a temperature of 25 °C was selected for all the experiments and a shear rate ramp from 0.2 to 250 s^−1^ was set for all the experiments. Then, the solutions rheological behavior was fitted to the Cross model (Equation (1)), determining the different parameters. $\eta_{0}$ is the zero-shear-rate viscosity, $\eta_{\infty }$ is the infinity shear viscosity, K is the consistency, and *m* is the rate constant. These parameters give information about the rheological characteristics of the solution. Specifically, *m* indicates the power law relation between shear stress and shear rate in the shear-thinning region (a value of *m* = 0 indicates a predominant Newtonian behavior) whereas K is related to the relaxation times (the reverse 1/K indicates the critical strain rate (${\dot{\gamma }}_{C}$)). In our case, since a Newtonian plateau at large velocities of deformation was not found, $\eta_{\infty }$ was assumed as 0 [29]

(1) $$\eta =\eta_{\infty }+\frac{\eta_{0}-\eta_{\infty }}{1+{\left(K\cdot \dot{\gamma }\right)}^{m}}$$

Then, oscillatory analyses (specifically frequency sweeps) were performed on the different gels and the different solutions in order to determine the storage (G′) and the loss modulus (G″). These moduli provide information about the gel/solution characteristics.

Frequency sweeps were performed for an angular frequency, from 0.5 rad/s within the linear range, which was determined after performing a strain sweep at a 1 Hz frequency. Frequency sweep also gives information about the values of G′and G″. This modulus can be also used to calculate the complex viscosity of the gels.

The value of G″ was also used to identify the effect of the Laponite^®^ in the yield, or critical, stress of different solutions. Specifically, with the modified Casson model (Equation (2))

(2) $$\sqrt{G^{\prime \prime }}=\sqrt{\sigma_{0}}+\sqrt{kw}$$

The previous equation shows the relationship between the loss modulus G″, the angular frequency *w*, the viscosity coefficient *k* and the yield stress *σ*_0_. The yield stress, minimun energy to break the polymer solutions, can be obtained after plotting G″^1/2^ against *w*^1/2^ (plot interception) as was done in [30].

The rheological measurements were performed after 2 days of samples (solutions or gels) preparation to compare the results at the same aging time.

### 3.4. Gel Formation

Hydrogels of chitosan and alginate were formed by crosslinking them with glutaraldehyde and calcium chloride, respectively. Firstly, an initial amount of the polymer and Laponite^®^ are solved and dispersed together in 20 mL of distilled water under a continuous stirring for 24 h. Different gels were formed during these experiences: Alginate concentration was kept still at 2.0% *w*/*w* (which equals 0.4 g), and Laponite^®^ concentrations were fixed at 0.5%, 1.0% and 2.0% *w*/*w* (using 0.1, 0.2 and 0.4 g, respectively). Chitosan gels were formed with a chitosan concentration of 1% *w*/*w* (0.2 g) with Laponite^®^ concentrations 0.5, 1.0 and 2.0% *w*/*w* (0.1, 0.2 and 0.4 g). It is important to add a few mL of acetic acid in to chitosan solutions in order to acidify pH and obtain a homogeneous phase. After that, a fixed amount of the crosslinker is added, also with vigorous stirring, to produce the gel.

Crosslinker agent for alginate solutions (CaCl_2_) was implied using a fixed proportion of 2.0 mL of CaCl_2_ (2.0% *w*/*w*) for 20 mL alginate solution (2.0% *w*/*w*), i.e., 2.0 mL of a 2.0% *w*/*w* CaCl_2_ solution were added to every alginate solution. Chitosan gels were, meanwhile, formed with crosslinker glutaraldehyde, adding 2 mL to each chitosan solution.

In this context, it should be specified that only gels of chitosan and alginate were produced, since levan cannot be gelled without performing surface modification, and xanthan gum is mainly used to produce films hydrogels by taking advantage of the transition between its helical and coil conformations.

## 4. Results and Discussion

### 4.1. Laponite^®^ Effect on Polysaccharides Solutions

Two different rheological analysis were performed: a steady flow test and an oscillatory test. Concentrations of 1.0, 3.0 and 5.0% *w*/*w* of the polysaccharide, with a variable Laponite^®^ concentration of 0.5, 1.0 and 2.0% *w*/*w* were studied. These Laponite^®^ concentrations were selected because according to different articles, a concentration of 2.0% *w*/*w* in water is essentialconcerning Laponite^®^ dispersions structure, as is indicated in [6,19]

#### 4.1.1. Steady Flow Test for Solutions of Polysaccharides

**Figure 1:**
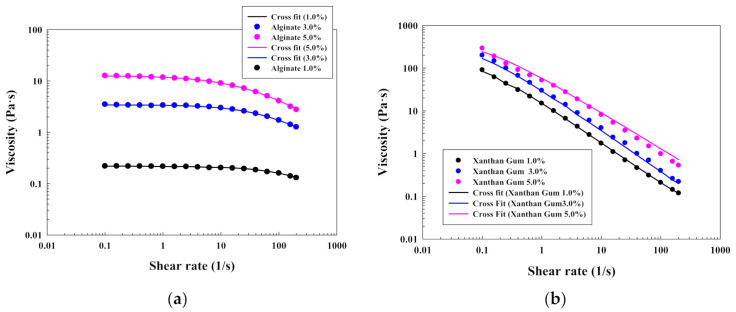
Shear stress-shear rate plots for solutions of (**a**) alginate; (**b**) xanthan gum.

First results are concerning the steady flow behavior of the different polymeric solutions with and without Laponite^®^. Figure 1a,b illustrates the results for the different polymeric solutions of alginate and xanthan gum, in terms of shear stress-shear rate with the Cross fit, without Laponite^®^. Results for chitosan and levan are included in the Supplementary Material (Figure S1). In this context, it should be specified that levan solutions in water (at all the investigated concentrations) have a constant viscosity with a Newtonian behavior, as was also obtained in [28].

First of all, the higher the concentration, the more shear-thinning behavior was found for all the investigated polymers without Laponite^®^ (non-Newtonian pseudoplastic behavior). This behavior is explained by taking into account the polymer swelling and the space reduction in the macromolecular structure. These facts increase the interactions inside the polymeric structure, causing an increase of the pseudoplasticity.

Furthermore, a progressive increase in the shear stress provokes the alignment of the different polymeric structures and the viscosity is reduced until reaching a Newtonian behavior. This phenomenon has been already reported for a high number of polymeric solutions, such as chitosan [31], and is illustrated in Figure 1a for alginate and Figure S1 (Supplementary Material) for chitosan. Moreover, the results can be explained with the parameters (Table 1) that were obtained after modelling the shear stress-shear rate with equation 1 (Cross model). Specifically, the behavior of the different solutions was studied by considering the zero-rate viscosity and the critical strain as key parameters. For all the investigated solutions, the higher the concentration, the higher the zero-rate viscosity for all the solutions. In this context, chitosan solutions have a larger zero-rate viscosity than the alginate ones.

At the same time, the critical strain rate, which gives information about the onset shear rate for shear thinning, increases with a decrease on the concentration. This phenomenon is predominantly observed for alginate (Figure 1a) and chitosan (Figure S1A, Supplementary Information) solutions because they have a clear initial plateau before reaching the shear thinning effect. However, xanthan gum solutions (Figure 1b) show a marked pseudoplastic effect already at low shear rates, without the existence of that initial plateau and, as a consequence, a shorter critical strain. That fact can be a consequence of the existence of a yield stress. This phenomenon can be observed in Supplementary Material (Figure S2) and has been previously reported in [32]. Moreover, this yield stress increase will be further evaluated in Section 4.1.2 with the modified Casson plot (Figure 6c).

The differences in the previous results are a consequence of the different polymeric structures of the investigated polymers and their ionic character. Levan forms nanoparticles in water by a self-assembly process, and xanthan gum has a structure with helix, whereas alginate and chitosan are mainly constituted by lineal polymeric chains with different functional groups and with different charge (alginate is anionic whereas chitosan is cationic).

**Figure 2:**
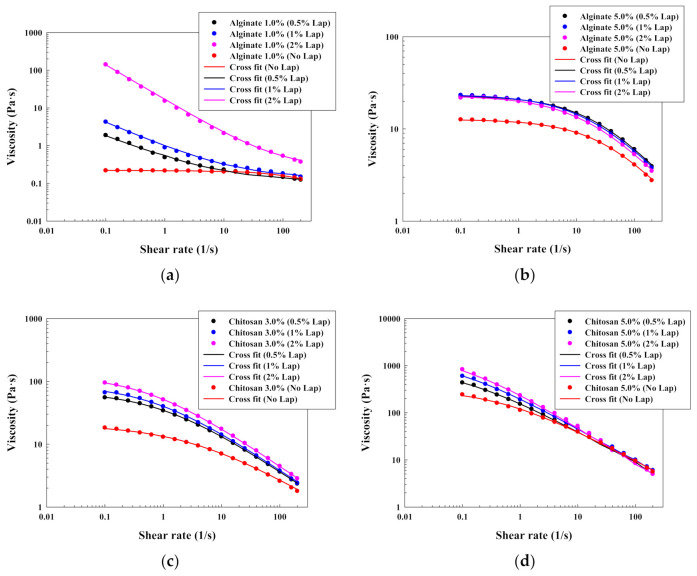
Viscosity-shear rate plots for different polysaccharides solutions with Laponite: (**a**) alginate 1.0% *w*/*w*; (**b**) alginate 5.0% *w*/*w*; (**c**) chitosan 3.0% *w*/*w*; (**d**) chitosan 5.0% *w*/*w*.

**Figure 3:**
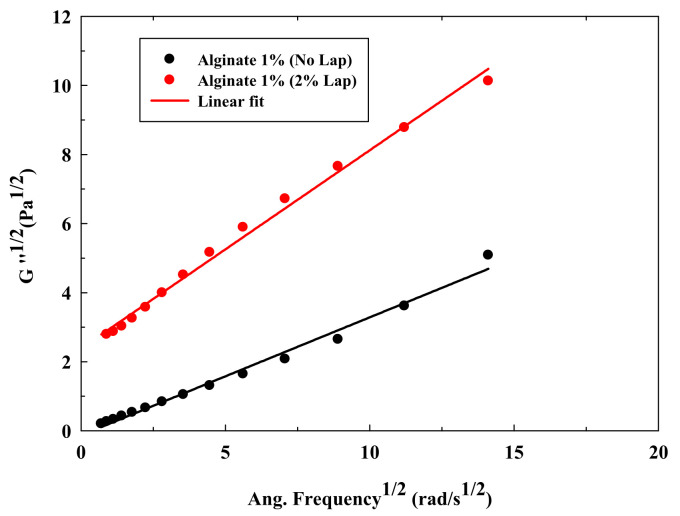
Modified Casson model for alginate solution (1.0% *w*/*w*) and alginate solution (1.0% *w*/*w*) with 2.0% *w*/*w* of Laponite^®^.

Alternatively, Figure 2a (alginate 1.0% *w*/*w*), Figure 2b (alginate 5.0% *w*/*w*), Figure 2c (chitosan 3.0% *w*/*w*) and Figure 2d (chitosan 5.0% *w*/*w*) illustrate the typical effect of the Laponite^®^ on the rheological behavior of polymeric solutions (lineal chains) of alginate and chitosan (results for alginate 3.0% *w*/*w* and chitosan 1.0% *w*/*w* can be found in Figure S3 in the Supplementary Material). Moreover, Table 2 shows the parameters that were obtained after fitting the data with the Cross model. In this case, it is possible to observe how, in general, Laponite^®^ increases the zero-rate viscosity for all the investigated solutions, probably due to the formation of a platelet structure via electrostatic interactions. However, the impact of the Laponite^®^ is stronger on solutions with a larger critical strain and a lower zero-rate viscosity. That fact can be observed with alginate at 1.0% *w*/*w* (Figure 2a). In this case, the addition of Laponite^®^ modifies drastically the rheological behavior of the polymeric solutions, reducing largely the critical strain and increasing the zero-rate viscosity. This significant increase in the zero-rate viscosity and consistency, at only this polymer concentration, is attributed to the change in the curve shape, capturing the Cross equation a large initial viscosity. This high zero-rate viscosity indicates a strong initial resistance to flow and the existence of a yield stress. Therefore, the modified Casson model plot (Figure 3) was built for the solutions at 1.0% *w*/*w* of alginate with Laponite^®^. This figure shows that the addition of Laponite^®^ produced an initial yield stress, mainly at 2.0% *w*/*w* of the clay, since the plot interception increases from 0 Pa^1/2^ (alginate without Laponite^®^) to 2.30 Pa^1/2^ (1.0% *w*/*w* alginate with 2.0% *w*/*w* Laponite^®^). Similar results were obtained by [19], and this phenomenon can be produced if the effect of the clay is predominant over the polymer entanglement, causing the Laponite^®^ structure this drastic change on the flow curve (mainly at 2.0% *w*/*w* because the platelet structure is more extended). However, this drastic effect was not found for alginate solutions at higher concentrations (Figure 2b). In this case, the polymer can reduce the electrostatic interactions and the possibility of the clay to form a platelet structure due to the polymer adsorption on the clay surfaces, and the polymeric behavior becomes predominant [19].

The same behavior was observed for chitosan solutions. The addition of Laponite^®^ reduces the critical strain (although for 5.0% *w*/*w* solutions the critical strain has a low and almost constant value) and without changing the pseudoplastic character of the polymeric solutions. Nevertheless, in spite of the increase on the zero-rate viscosity due to the clay addition, it is important to highlight that the chitosan solutions without Laponite^®^ have a larger zero-rate viscosity in water than the alginate ones. As a consequence, the effect of the Laponite^®^ in the flow curve only modifies the solution parameters, without changing the shape of the shear flow curve, as can be seen in Figure 2c,d.

**Figure 4:**
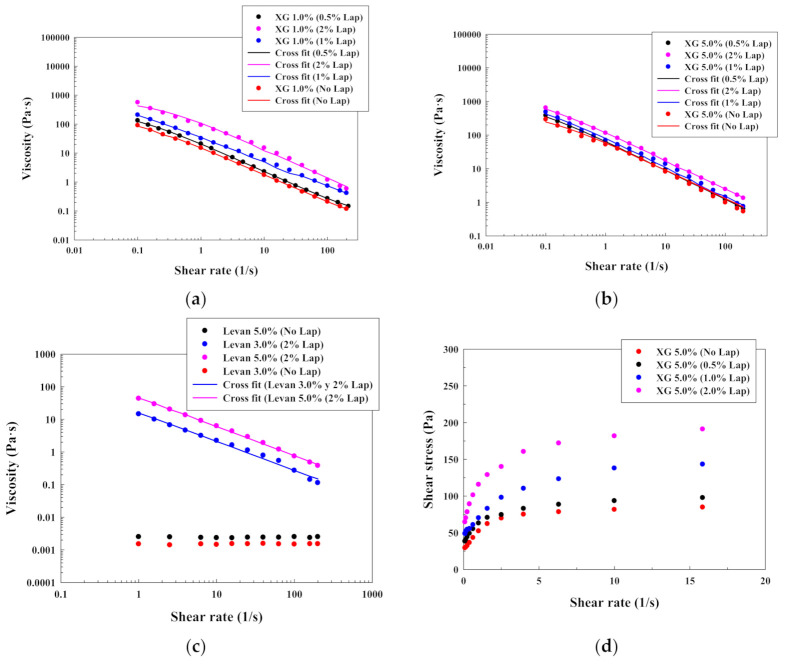
Viscosity-shear rate plots for different polysaccharides solutions with Laponite^®^: (**a**) xanthan gum 1.0% *w*/*w*; (**b**) xanthan gum 5.0% *w*/*w*; (**c**) levan with 2.0% *w*/*w* Laponite^®^; (**d**) shear stress–shear rate plot for xanthan gum 5.0% *w*/*w* at low shear rates.

The obtained results concerning the effect of Laponite^®^ in xanthan gum (helical conformation with yield stress) and levan (nanoparticles formation by self-assembly phenomenon) solutions are illustrated in Figure 4a (xanthan gum 1.0% *w*/*w*), Figure 4b (xanthan gum 5.0% *w*/*w*), Figure 4c (levan with 2.0% Laponite^®^) and Table 2 (Cross model parameters). Results for xanthan gum 3.0% *w*/*w* are included in the supplementary material (Figure S4).

As happened with the alginate and chitosan, the addition of Laponite^®^ increased the zero-rate viscosity whereas the critical strain of the xanthan gum solutions was low and almost constant, probably due to the high zero-rate viscosity of the solutions without Laponite^®^. Again, the effect of the Laponite^®^ does not change the shape of the shear flow curve (Figure 4a,b). Furthermore, as can be seen in Figure 4d, the addition of Laponite^®^ increases the yield stress of the xanthan gum solutions due to the existence of a more ordered structure. These results were also checked with the modified Casson model in Section 4.1.2 (Figure 6c).

The effect of the Laponite^®^ was also studied with levan, which forms Newtonian solutions in water for all the investigated concentrations, due to its amphiphilic properties. In this case, similar results were obtained in [28], in which they showed that levan formed Newtonian solutions up to a concentration of 30% *w*/*w*. However, a significant effect was observed when a concentration of 2.0% *w*/*w* of Laponite^®^ was added into solutions of 3.0% *w*/*w* and 5.0% *w*/*w* of levan (Figure 4c). Here, Laponite^®^ promoted a marked shear-thinning effect without the existence of an initial plateau that could be also checked when the results were modelled with Cross model. This effect is produced due to the existence of a platelet structure, which is modified due to the possible interactions between levan nanoparticles and the clay. In this context, nanoparticles of levan have a size of 150 nm (Figure S5 in Supplementary Material) and a zeta potential of around −2.0 mV (Figure S6 in Supplementary Material).

Previous results indicate how the effect of the Laponite^®^ in polymeric solutions depends on properties such as polymer amphiphilicity, zero-rate viscosity, polymer charge or even the conformation. In general, Laponite^®^ provides a higher impact on low concentrated polymeric solutions with a low zero-rate viscosity. In this case, the solutions behavior can be drastically modified due to the addition of a platelet structure, increasing the shear-thinning effect, the zero-rate viscosity and including a yield stress to the polymeric solution. However, although increasing also the zero-rate viscosity, the Laponite^®^ impact on the shape of the shear flow curve is reduced for high concentrated polymeric solutions with a marked shear-thinning effect. Moreover, the addition of Laponite^®^ provides also a strong modification of the character of a polymeric solution with nanoparticles that were formed by self-assembly.

#### 4.1.2. Oscillatory Flow Tests for Solutions of Polysaccharides

**Figure 5:**
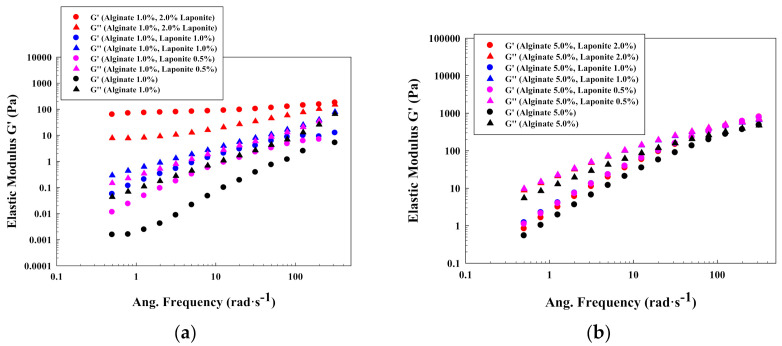

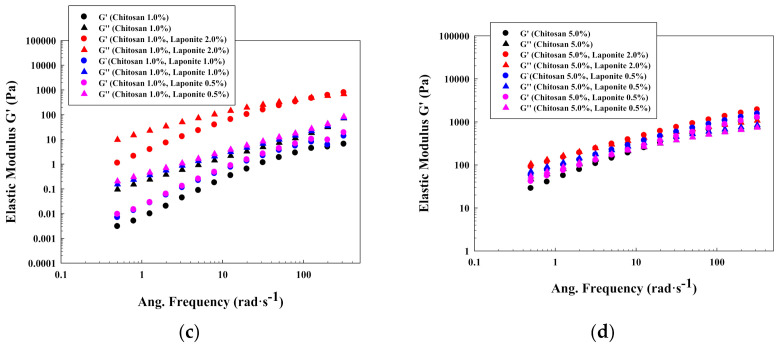
Oscillatory plots for different polysaccharides solutions: (**a**) alginate 1% *w*/*w*; (**b**) alginate 5% *w*/*w*; (**c**) chitosan 1% *w*/*w*; (**d**) chitosan 5% *w*/*w*.

As was done for the steady shear flow in the previous section, oscillatory results were obtained for the different systems with and without Laponite^®^. First of all, it has been confirmed that polysaccharides concentrations in the solutions do affect the system properties. It was demonstrated that when polysaccharide concentrations increased, both elastic and viscous modulus increased (Figure 5a–d).

Alginate is shown to be a viscous exopolysaccharide, due to its liquid-like behavior determined by its viscous modulus with a higher value than its elastic modulus when no Laponite^®^ interferes. However, with increasing Laponite^®^ concentrations, the liquid nature is less evident, since elastic modulus increases, even up to a point where Laponite^®^ concentration overcomes alginate concentration, and the solution is found to be predominantly elastic (Figure 5a). This phenomenon is caused due to the existence of a more ordered structure that predominates to achieve a solid-like behavior, providing long time terms in the relaxation time distributions.

However, when alginate concentration is increased, this phenomenon gets more complicated to achieve due to its increase in the chain entanglement (Figure 5b for an alginate concentration of 5.0% *w*/*w*), which avoids the formation of the platelet structure. These phenomena agree with the obtained data for the steady flow. In these cases, the mechanical spectra are similar at all the investigated conditions, resembling the typical behavior of polymeric solutions, and as consequence, the structure of the polymeric chains is predominant. Data for alginate 3.0% *w*/*w* is illustrated in Supplementary Material (Figure S7a).

The typical behavior of polymeric solutions was also found for low concentrated chitosan solutions. In this case, the addition of the clay did not modify significantly the nature of the solutions (Figure 5c). The similarities that were found between the behavior for high concentrated alginate solutions and low concentrated chitosan solutions are attributed to the different character of the polymeric solutions. According to Table 2, chitosan solutions showed larger zero-rate viscosity and shorter critical strain rates. The addition of Laponite^®^ had a less significant effect in this type of solutions because the polymeric entanglement predominated.

Finally, the addition of Laponite^®^ on high concentrated solutions in chitosan did not have an important effect on the mechanical spectrum, since always similar elastic and viscous modulus were found, predominating the polymer entanglement (Figure 5d). Data for chitosan 3.0% *w*/*w* is included in Supplementary Material (Figure S7b).

**Figure 6:**
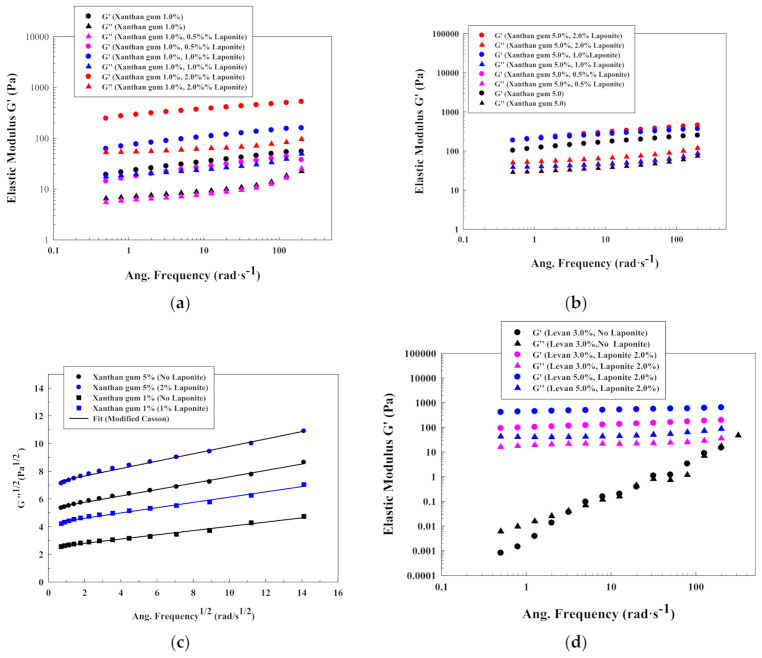
Oscillatory plots for different polysaccharides solutions: (**a**) xanthan gum 1.0% *w*/*w*; (**b**) xanthan gum 5.0% *w*/*w*; (**c**) modified Casson model for xanthan gum solutions; (**d**) Levan.

Xanthan gum is a completely different case, due to the elastic behavior that present all the solutions studied (even without Laponite^®^). In this case, Laponite^®^ (when added in a concentration above 1.0% *w*/*w*) promotes an increase on the elasticity due to the clay-polymer macromolecular interactions, restricting again chains mobility (Figure 6a,b). This increase in elasticity is stronger for 1.0% *w*/*w* solutions of xanthan gum because the polymer entanglement does not interfere with the formation of a structure due to the addition of Laponite^®^. Again, this result agrees with the steady flow experiments. Moreover, there is an increase in the elastic modulus of the system with the frequency, indicating that the structure resembles a weak gel. Moreover, Figure 6c shows the modified Casson plot for different xanthan gum solutions with different amounts of Laponite^®^. In this case, it is possible to observe how the curve intercepts the axis at different points, highlighting the change in the xanthan gum yield stress due to the addition of the inorganic solid.

Levan presents a different behavior as well. Its rheological behavior is shown to be close to liquid-like materials (an example with a solution of 3.0% *w*/*w* levan is illustrated in Figure 6d). Nevertheless, the addition of a 2.0% *w*/*w* of Laponite^®^ increases significantly the elastic nature of the system, and it behaves as a weak gel structure. In this case, the addition of the clay predominates over the polymer concentration due to the amphiphilic polymer properties (Figure 6d).

The previous results indicate that the character of the initial polymeric solutions (existence of a yield stress, amphiphilic properties, pseudoplasticity) is also important concerning the effect of the Laponite^®^ in solutions’ elastic or viscous nature. As was observed with the steady shear flow results, the Laponite^®^ had a significant effect on high Newtonian solutions, as happened with alginate at low concentrations or levan. In this case, the solutions can get a more solid nature because the Laponite^®^ can confer a more stable and elastic structure, by increasing the relaxation times distribution that predominates over the polymer entanglement.

However, for more concentrated or more viscous solutions (alginate at high concentration or chitosan), the Laponite^®^ did not have a significant effect, without modifying the typical mechanical spectra of polymeric solutions.

Nevertheless, that fact was not observed for xanthan gum solutions (coil conformation with an initial yield stress), in which the oscillatory analysis always indicated the existence of a gel-like structure (Figure 5a,b). In this case, the Laponite^®^ increased the elastic modulus, providing increasing the elastic behavior to the solution.

### 4.2. Alginate and Chitosan Gels

**Figure 7:**
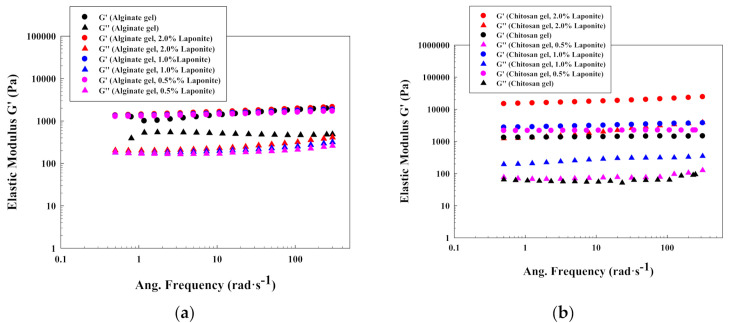
Oscillatory plots with the effect of Laponite^®^ on (**a**) alginate gels; (**b**) chitosan gels.

The last section explains the effect of the addition of Laponite^®^ in alginate and chitosan gels, at 2.0% *w*/*w* of alginate and 1.0% *w*/*w* of chitosan (Figure 7). These concentrations were selected because numerous articles produced gels from these concentrations.

For both systems, the elastic nature of the gels clearly predominates over the viscous one, and the elastic modulus is constant with the frequency, indicating gel stability. However, the effect of Laponite^®^ is different for alginate and chitosan.

Specifically, the addition of Laponite^®^ did not have an important effect on these alginate gels (Figure 7a) at all the investigated conditions (from 0.0% to 2.0% *w*/*w*). That fact indicates that the interactions between the Laponite^®^ platelets, at this concentration, that are included in the formed gel network are weak and not enough for reducing the intramolecular space inside the backbone to increase the elasticity of this hydrogel (elastic modulus always around 2000 Pa). On the other hand, Laponite^®^ increased significantly the elastic modulus of chitosan gels (Figure 7b) at a concentration of 2.0% *w*/*w* (from 1000 to 20,000 Pa).

Therefore, these results may indicate that the effect of Laponite^®^ on chemical gels (covalent bond) is dependent on the ionic nature of the polymers and their structure and, as a consequence, also dependent on the crosslinking mechanism. In this case, due to the different nature of the initial polymers (cationic chitosan and anionic alginate), the crosslinking reaction varies. Chitosan requires the protonation of ammonia and the chitosan uses the carboxylic acid functional groups. Moreover, Laponite^®^ has negative and positive charges in its structure. Therefore, the interactions with the different functional groups can be different, and as a consequence, the elastic modulus value is also different.

These results also point out that the Laponite^®^ can be added to different gels to modify the elasticity of the material. This fact can be useful for modifying hydrogels’ structure depending on the application.

## 5. Conclusions

The impact of the addition of the Laponite^®^ on the rheological behavior of different polysaccharides (alginate, chitosan, xanthan gum and levan) has been investigated in this work. In general, the initial parameters (zero-rate viscosity and critical strain rate) of the solutions define the effect of the clay. Although Laponite^®^ always increased the shear-thinning behavior of the solutions, this increase was different depending on the solutions’ ideality. Polymeric solutions with high zero-rate viscosity and short critical strain rates were predominant over the Laponite^®^ effect, being independent on the polymer structure. Alternatively, the Laponite^®^ effect was predominant for low polymer concentrations, providing a significant increase on the zero-rate viscosity and also decreasing the critical strain rates as well as including an initial resistance to flow (yield stress). This phenomenon is attributed to the formation of a more ordered platelet structure. Particularities were found for polymeric solutions with an initial yield stress (xanthan gum) and with amphiphilic properties (levan). In these cases, Laponite^®^ increased the initial yield stress and had a drastic effect on the nanoparticle solutions if the clay conforms an ordered structure.

A similar trend was observed for the oscillatory analysis. Laponite^®^ had a stronger effect on solutions with a low zero-rate viscosity or with a Newtonian behavior (levan colloidal solutions), and a physical gelation can be produced with a high concentration of the clay.

Although Laponite^®^ always increased the elastic modulus, that effect was less important for solutions of chitosan and alginate with short critical strain rate and large zero-rate viscosity, always keeping the typical mechanical spectra for polymeric solutions. Finally, it was checked how the addition of Laponite^®^ modified typical gels of alginate and chitosan. The effect was stronger for chitosan gels probably due to the existing differences between the crosslinking reaction, which involve a protonation and positive charges that can interact in a greater extent with the clay negative charges.

## Figures and Tables

**Table 1:** Cross fit results (no Laponite^®^).

Solution	Zero-Rate Viscosity (Pa·s)	Consistency (s)	Critical Strain (s^−1^)	Rate Index	Standard Error
Alginate (1.0% *w*/*w*)	0.22	3.10 × 10^−3^	333.33	0.84	14.43
Alginate (3.0% *w*/*w*)	3.42	1.20 × 10^−2^	83.33	0.86	6.59
Alginate (5.0% *w*/*w*)	12.66	2.80 × 10^−2^	35.71	0.73	5.35
Chitosan (1.0% *w*/*w*)	0.29	7.80 × 10^−3^	128.20	0.25	83.36
Chitosan (3.0% *w*/*w*)	20.34	0.31	3.22	0.55	7.06
Chitosan (5.0% *w*/*w*)	314.80	2.09	0.48	0.65	11.06
Xanthan Gum (1.0% *w*/*w*)	199.60	13.69	0.07	0.96	5.30
Xanthan Gum (3.0% *w*/*w*)	399.01	14.01	0.07	0.96	8.12
Xanthan Gum (5.0% *w*/*w*)	517.20	11.36	0.09	0.88	14.16

**Table 2:** Cross fit results (with Laponite^®^).

Solution	Laponite^®^ Concentration % *w*/*w*	Zero-Rate Viscosity (Pa·s)	Consistency (s)	Critical Strain (s^−1^)	Rate Index	Standard Error
Alginate (1.0% *w*/*w*)	0.5	4.43 × 10^3^	3.11 × 10^6^	3.21 × 10^−7^	0.62	20.09
	1.0	21.39 × 10^3^	3.59 × 10^6^	2.80 × 10^−7^	0.67	16.17
	2.0	1.25 × 10^6^	2.00 × 10^5^	5.00 × 10^−6^	0.92	8.55
Alginate (3.0% *w*/*w*)	0.5	5.39	1.60 × 10^−2^	62.50	0.82	4.69
	1.0	5.82	2.20 × 10^−2^	45.45	0.65	8.12
	2.0	11.06	0.17	5.88	0.45	25.92
Alginate (5.0% *w*/*w*)	0.5	22.92	4.22 × 10^−2^	23.81	0.72	5.76
	1.0	22.82	5.21 × 10^−2^	19.23	0.69	8.29
	2.0	23.62	5.96 × 10^−2^	16.77	0.68	8.08
Chitosan (1.0% *w*/w)	0.5	0.30	7.30 × 10^−3^	136.98	0.39	44.12
	1.0	0.37	9.60 × 10^−3^	104.17	0.61	28.02
	2.0	0.49	1.20 × 10^−2^	83.33	0.71	22.17
Chitosan (3.0% *w*/*w*)	0.5	69.11	0.99	1.01	0.63	3.03
	1.0	89.41	1.39	0.72	0.63	5.36
	2.0	126.10	1.77	0.56	0.64	2.75
Chitosan (5.0% *w*/*w*)	0.5	888.30	10.56	0.09	0.66	5.53
	1.0	1.38 × 10^3^	14.48	0.07	0.68	5.63
	2.0	1.53 × 10^3^	9.19	0.10	0.75	8.67
Xanthan Gum (1.0% *w*/*w*)	0.5	298.40	14.31	0.07	0.98	4.20
	1.0	666.10	55.55	1.80 × 10^−2^	0.98	23.00
	2.0	1.15 × 10^3^	62.75	1.60 × 10^−2^	0.84	8.15
Xanthan Gum (3.0% *w*/*w*)	0.5	1.09 × 10^3^	24.18	0.04	0.93	10.20
	1.0	1.17 × 10^3^	102.60	0.01	0.85	11.80
	2.0	1.16 × 10^3^	50.84	0.02	1.00	25.38
Xanthan Gum (5.0% *w*/*w*)	0.5	1.20 × 10^3^	27.32	0.04	0.87	7.19
	1.0	1.35 × 10^3^	24.44	0.04	0.87	19.41
	2.0	1.84 × 10^3^	23.62	0.04	0.85	6.78
Levan (2.0% *w*/*w* Laponite^®^)	3.0%*w*/*w* Levan	258.90	21.88	0.05	0.89	21.95
	5.0%*w*/*w* Levan	509.30	13.55	0.07	0.90	8.88

## Data Availability

This study does not report any data.

## Supplementary Materials

The following are available online at https://www.mdpi.com/2073-4360/13/6/966/s1, Figure S1: Viscosity-shear rate plot for (a) chitosan solution and (b) levan solutions, Figure S2: Shear stress-shear rate plot for xanthan gum solutions (low shear rate results) Figure S3: Viscosity-shear rate plot for (a) alginate at 3.0% *w*/*w* and (b) chitosan at 1.0% *w*/*w*, Figure S4: Viscosity-shear rate plot for xanthan gum (3.0% *w*/*w*), Figure S5: TEM images of levan nanoparticles, Figure S6: Z potential of levan nanoparticles. Figure S7: Oscillatory results for (a) alginate 3.0% and (b) chitosan 3.0%.
