# Supplementary material for: Steady and Oscillatory Shear Flow Behavior of Different Polysaccharides with Laponite®
Source: Polymers (Basel). 2021 Mar 22;13(6):966. doi: 10.3390/polym13060966 (PMC8004235; doi:10.3390/polym13060966)
Supplement: Supplementary file 1 [file polymers-13-00966-s001.pdf]

polymers

MDPI

Supplementary material

Steady and Oscillatory Shear Flow Behavior of Different Polysaccharides with Laponite®

Marcos Blanco-López [1], Álvaro González-Garcinuño [1], Antonio Tabernero [1,*] and Eva M. Martín del Valle [1,2,*]

[1] Department of Chemical Engineering, University of Salamanca, Plaza los Caídos s/n, 37008 Salamanca (SA), Spain; marcosbl97@usal.es (M.B.L.), alvaro_gonzalez@usal.es (A.G.-G.)

[2] Instituto de Investigación Biomédica de Salamanca, Hospital Virgen de la Vega, Paseo San Vicente, 58-182, 37007 Salamanca (SA), Spain;

* Correspondence: antaber@usal.es (A.T.); emvalle@usal.es (E.M.d.V.)

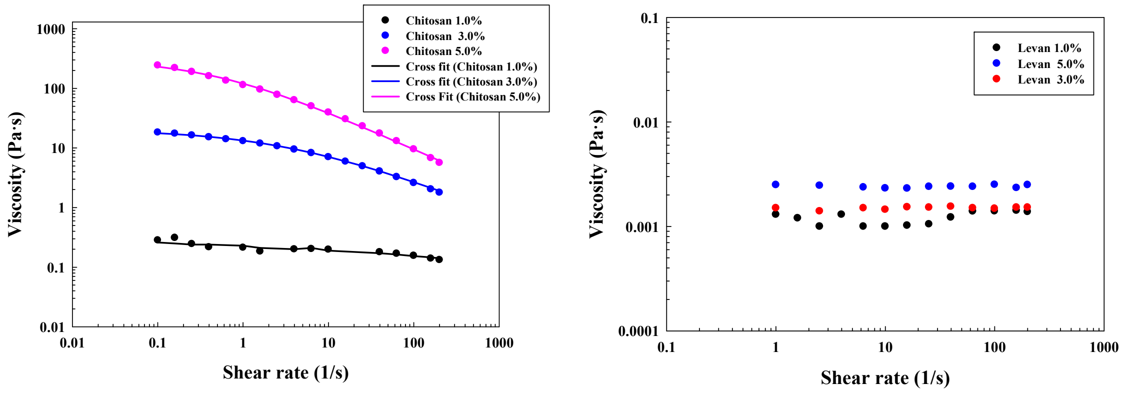

(**a**) (**b**)

Figure S1. Viscosity-shear rate plot for **a**) chitosan solution and **b**) levan solutions.

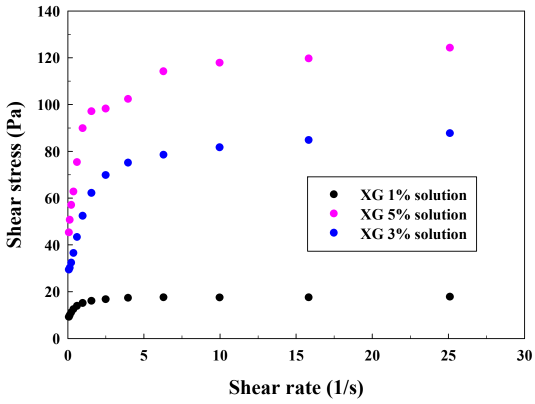

Figure S2. Shear stress-shear rate plot for xanthan gum solutions (low shear rate results).

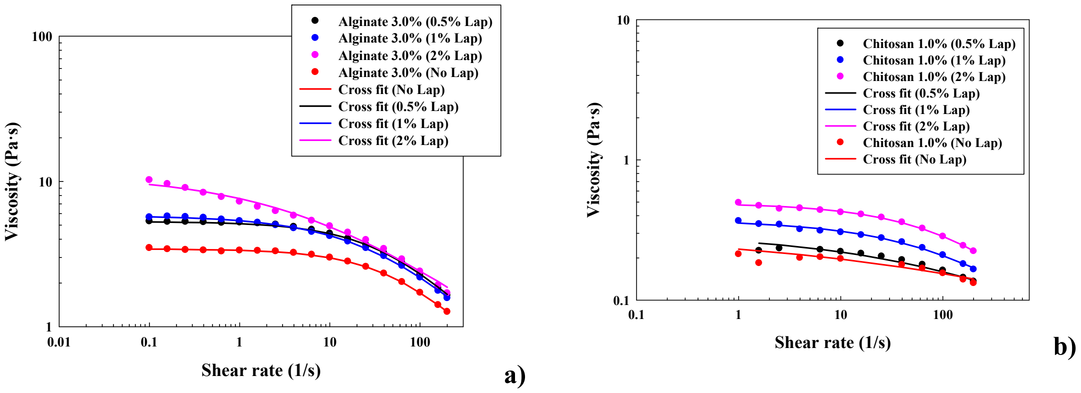

Figure S3. Viscosity-shear rate plot for **a**) alginate at 3.0% *w*/*w* and **b**) chitosan at 1.0% *w*/*w*.

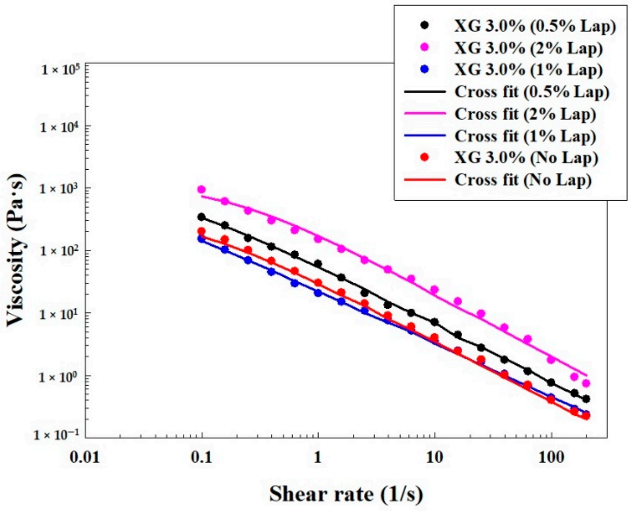

Figure S4. Viscosity-shear rate plot for xanthan gum (3.0% *w*/*w*).

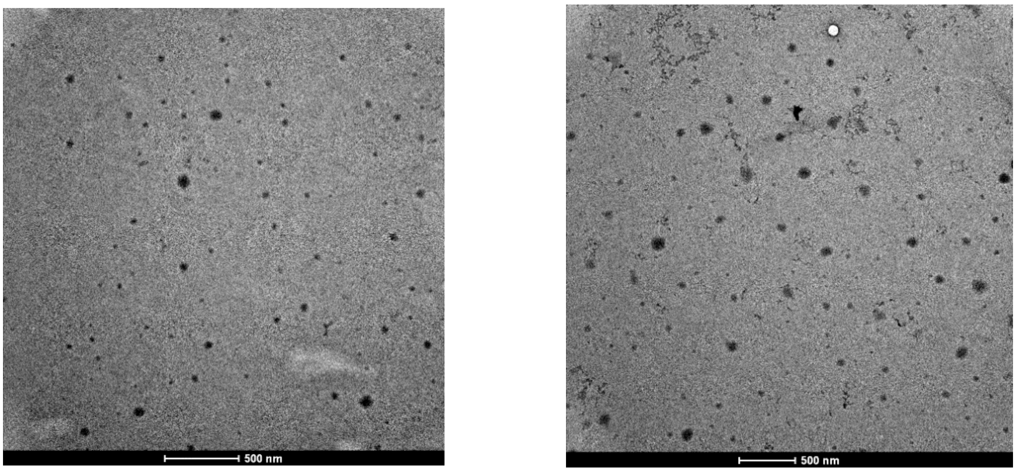

Figure S5. TEM images of levan nanoparticles.

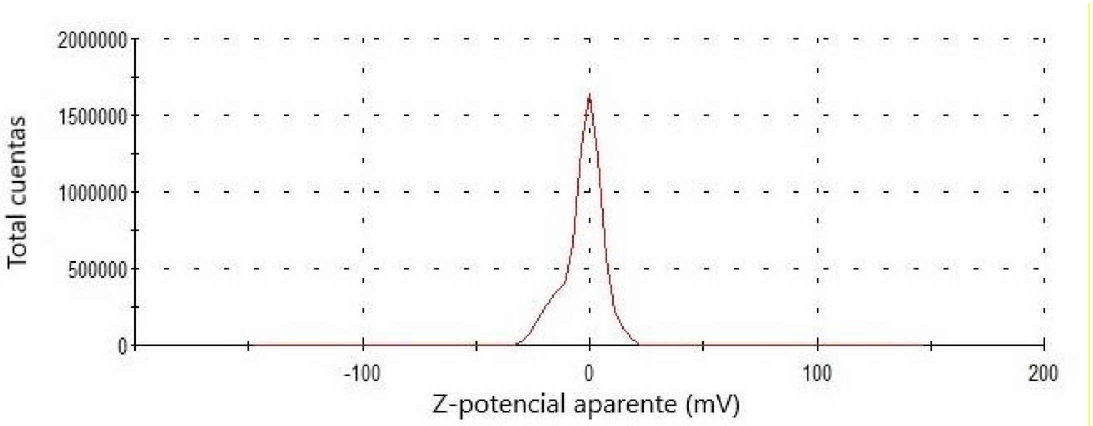

Figure S6. Z potential of levan nanoparticles.

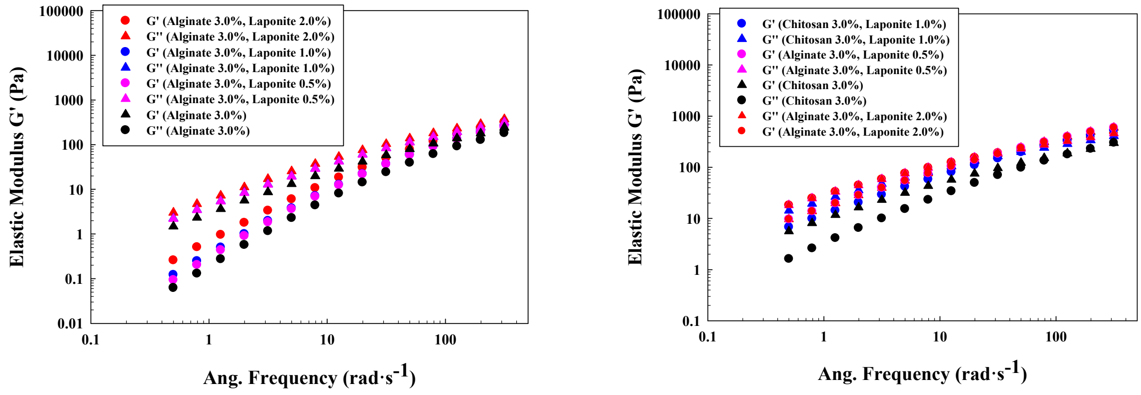

a)

Figure S7. Oscillatory results for **a**) alginate 3.0% and **b**) chitosan 3.0%.
